# Myeloid cell modulation by a GLP-1 receptor agonist regulates retinal angiogenesis in ischemic retinopathy

**DOI:** 10.1172/jci.insight.93382

**Published:** 2021-12-08

**Authors:** Lingli Zhou, Zhenhua Xu, Yumin Oh, Rico Gamuyao, Grace Lee, Yangyiran Xie, Hongkwan Cho, Seulki Lee, Elia J. Duh

**Affiliations:** 1 Wilmer Eye Institute and; 2 The Russell H. Morgan Department of Radiology and Radiological Sciences, Johns Hopkins University School of Medicine, Baltimore, Maryland, USA.

**Keywords:** Ophthalmology, Retinopathy

## Abstract

Ischemic retinopathies including diabetic retinopathy are major causes of blindness. Although neurons and Müller glia are recognized as important regulators of reparative and pathologic angiogenesis, the role of mononuclear phagocytes (MPs) — particularly microglia, the resident retinal immune cells — is unclear. Here, we found MP activation in human diabetic retinopathy, especially in neovessels from human neovascular membranes in proliferative retinopathy, including TNF-α expression. There was similar activation in the mouse oxygen-induced retinopathy (OIR) model of ischemia-induced neovascularization. Glucagon-like peptide-1 receptor (GLP-1R) agonists are in clinical use for glycemic control in diabetes and are also known to modulate microglia. Herein, we investigated the effect of a long-acting GLP-1R agonist, NLY01. Following intravitreal administration, NLY01 selectively localized to MPs in retina with OIR. NLY01 modulated MPs but not retinal endothelial cell viability, apoptosis, and tube formation in vitro. In OIR, NLY01 treatment inhibited MP infiltration and activation, including MP expression of cytokines in vivo. NLY01 significantly suppressed global induction of retinal inflammatory cytokines, promoted reparative angiogenesis, and suppressed pathologic retinal neovascularization. Collectively, these findings indicate the important role of mononuclear phagocytes in regulation of retinal vascularization in ischemia and suggest modulation of MPs as a potentially new treatment strategy for ischemic retinopathies.

## Introduction

Ischemic retinopathies, including diabetic retinopathy (DR), retinal vein occlusions, and retinopathy of prematurity, are leading causes of blindness globally. Capillary dropout and lack of revascularization characterize these diseases, with consequent upregulation of proangiogenic growth factors by ischemic retina that stimulates pathologic preretinal angiogenesis. A proinflammatory state, including upregulation of cytokines such as TNF-α, is thought to impair reparative angiogenesis in ischemic retina (1, 2). There is a great need for further understanding of the mechanisms regulating the retinal milieu and vasculature in ischemia.

In recent years, investigation has intensified with regard to nonvascular cellular elements of the neurovascular unit that regulate the retinal vasculature (3). Although the role of Müller glia (4, 5) and neuronal elements (6, 7) has become appreciated, relatively less is known about the role of immune cells in retinal neovascularization (NV). Mononuclear phagocytes (MPs), such as microglia and monocyte-derived macrophages (hereafter referred to as microglia/macrophages), are increasingly being linked to ischemic retinopathies (8). Indeed, a pivotal study implicated myeloid cells in regulation of ischemia-induced retinal angiogenesis. Specifically, neuropilin-1–positive MPs exacerbated pathologic NV in a mouse model of ischemic retinopathy (9).

Microglia are becoming increasingly appreciated for their role in retinal homeostasis and disease (10, 11). As the resident immune cells in the retina, microglia play an important role in retinal homeostasis, including maintenance of synaptic function (12). On the other hand, activated microglia can have pathogenic effects, as in photoreceptor degeneration, for instance (13). Microglial function with respect to retinal blood vessels has been less clear. Neuropilin-1–expressing microglia were found to be dispensable for retinal angiogenesis during development (14). A recent study demonstrated that microglia are the dominant myeloid cell population in a mouse model of ischemic retinopathy, both in the ischemic retina and in the pathologic neovascular tufts, with only rare blood-derived macrophages (15). However, the functional role of microglia in ischemic retinopathies and pathologic retinal angiogenesis has remained elusive. In this regard, microglial modulation studies could provide valuable insights into microglial function in this disease context.

Glucagon-like peptide-1 receptor (GLP-1R) agonists, including exenatide, are in common clinical use for reducing hyperglycemia in type 2 diabetes. Beyond their benefits for glycemic control, multiple studies have demonstrated that these drugs can target microglia and modulate their neuroinflammatory phenotype, with beneficial effects on the CNS in experimental models (16–21) as well as on retinal neurodegeneration in experimental diabetes (22). Strikingly, exenatide has exhibited beneficial effects on motor scores in a clinical trial of patients with Parkinson’s disease (19). In the retina, an important study demonstrated a beneficial effect of topical GLP-1R agonist treatment on neurodegeneration (22). In addition, exenatide treatment significantly reduced blood-retina barrier breakdown and retinal inflammatory gene expression in mice subjected to retinal ischemia/reperfusion injury, as well as direct modulation of cultured microglia (23).

Our group has investigated NLY01, a pegylated exenatide peptide and long-acting GLP-1R agonist with extended half-life in nonhuman primates (18). Previously, we reported that NLY01 exhibited efficient penetration of the blood-brain barrier. In mouse models of Parkinson’s disease, subcutaneously injected NLY01 protected against dopaminergic neuronal loss by direct modulation of brain microglia, leading to antiinflammatory and neuroprotective effects without any adverse events (18).

In the present study, we were interested in further elucidating the role of microglia in ischemic retinal disease, with respect to both reparative angiogenesis and pathologic preretinal NV. In tissue specimens from both human DR and the mouse model of oxygen-induced retinopathy (OIR), there was strong localization of microglia/macrophages in ischemic retina, including neovascular tufts. We investigated the direct effect of NLY01 in modulating retinal microglia, both in culture and OIR. Modulation of MPs with NLY01 suppressed its neuroinflammatory phenotype, improved reparative angiogenesis, and suppressed preretinal NV in OIR. These results indicate an important role for microglia/macrophages in regulating retinal angiogenesis and provides a new therapeutic strategy for DR and other ischemic retinopathies.

## Results

### MPs are activated in ischemic retina and associated with pathologic neovessels in human DR and a rodent model of ischemic retinopathy.

**Figure 1:**
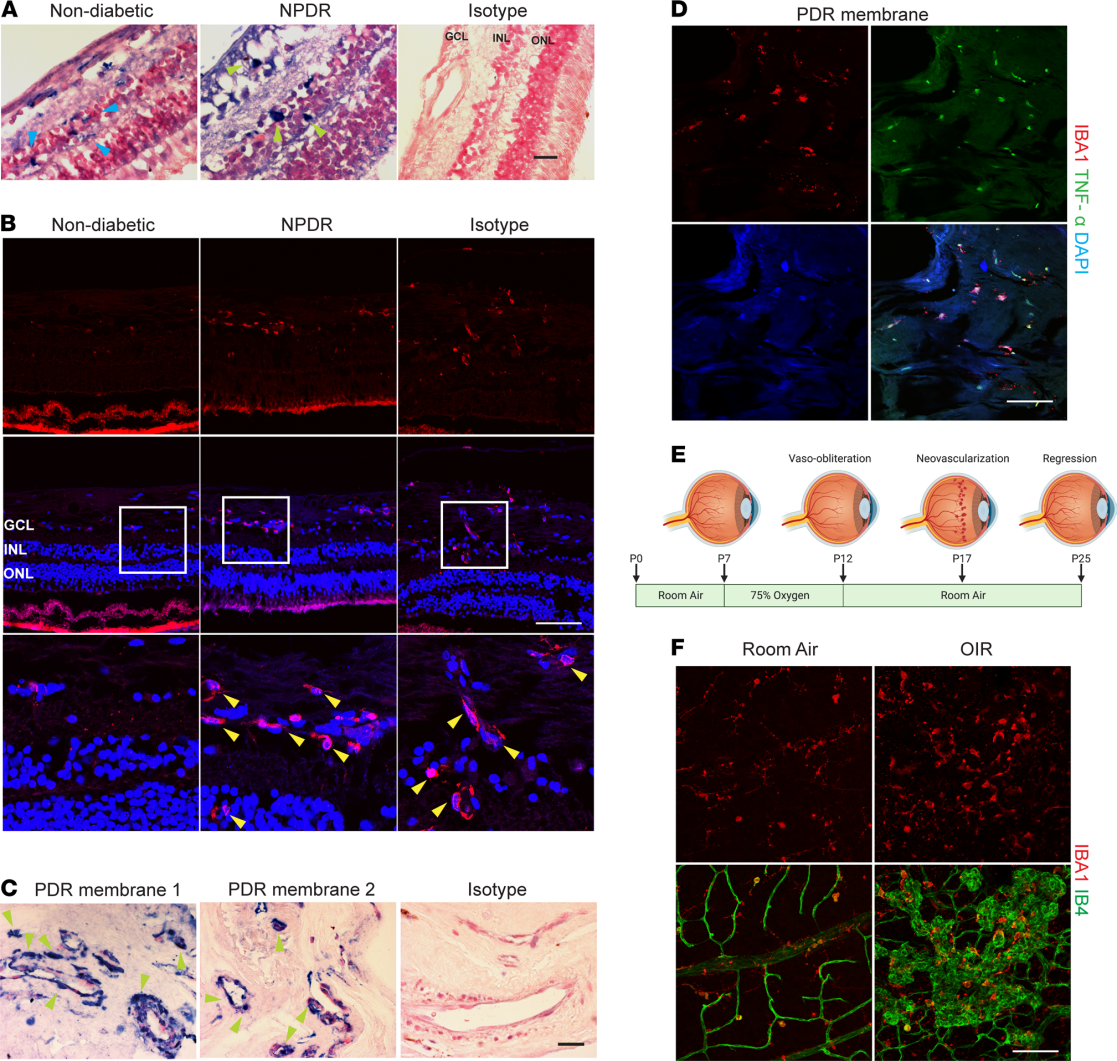
Microglial/macrophage activation in human diabetic retina, PDR membrane, and mice OIR retina. (**A**) MPs were immunostained with IBA1 antibody (blue) in nondiabetic and NPDR human eyes. In nondiabetic retina, ramified microglia (blue arrowhead) are presented in inner retinal layers. Hypertrophic MPs (green arrowhead) were observed in NPDR retina. *n* = 3. Scale bar: 100 μm. (**B**) Representative images of immunofluorescence (IF) staining of microglia/macrophages (red) in nondiabetic (left), NPDR (middle), and PDR (right) human eyes. In the nondiabetic control cases, IBA1 staining was weak. There were significantly more amoeboid microglia (yellow arrowheads) in both NPDR and PDR retinas. Blue: DAPI staining. (*n* = 6, nondiabetic eyes; *n* = 5 NPDR eyes; *n* = 6 PDR eyes). Scale bar: 100 μm. (**C**) Representative images of IHC staining of IBA1^+^ microglia/macrophages (blue) in membranes from patients with active PDR. Images were taken using a ×63 objective. Nuclear fast red was used for counterstaining. *n* = 3. Scale bar: 100 μm. (**D**) Representative IF staining of MPs (red) and TNF-α (green) in PDR membranes. Blue: DAPI staining. Scale bar: 100 μm. (**E**) Schematic diagram of OIR. Neonatal mice were exposed to 75% oxygen from P7 to P12 to induce vaso-obliteration and returned to room air from P12 to P17 to induce physiological revascularization and pathological NV, which reached maximum at P17. (**F**) Representative IF images of vasculature (IB4, red) and IBA1^+^ MPs (green) in retinal flat mounts from P17 OIR and room air (*n* = 3 mice). Scale bar: 100 μm.

In postmortem specimens, microglia are increased around the vasculature in nonproliferative diabetic retinopathy (NPDR), as indicated by staining for CD45 and CD68 (24), although human proliferative diabetic retinopathy (PDR) has not been examined. We first investigated the presence of microglia/macrophages in postmortem human specimens of nondiabetic retina and NPDR, using immunohistochemical analysis with IBA1, which is a more specific marker for detecting both quiescent and activated MPs in the CNS (25), and has been a useful marker of microglia in retinal studies, including retinal disease models (13, 26, 27). In nondiabetic retinal cross-sections, there were ramified IBA1^+^ microglia with small cell bodies in the ganglion cell layer and plexiform layers (Figure 1A), consistent with microglia in a quiescent state. In contrast to nondiabetic retina, IBA1^+^ MPs in NPDR retina had enlarged cell bodies.

We further investigated the presence of microglia/macrophages in nondiabetic, NPDR, and PDR cases with immunofluorescence staining. As shown in Figure 1B, there was a strong increase in amoeboid microglia/macrophages (yellow arrowheads) in both NPDR and PDR cases compared with the nondiabetic control, which had weak staining. The presence of these amoeboid-like microglia/macrophages is consistent with findings from a previous study (24) and suggests that microglial/macrophage activation is associated with NPDR even before progression to the more advanced stage of PDR, characterized by the presence of pathologic retinal NV.

We next examined the presence and localization of MPs in neovascular membranes from patients with PDR. Amoeboid MPs were prominent and robustly associated with the neovessels in PDR membranes (Figure 1C), suggesting interaction of activated microglia/macrophages with the neovasculature. We also found that the IBA1^+^ cells strongly expressed TNF-α (Figure 1D), an important marker of microglial activation (28) and a key pathogenic cytokine in ischemic retinopathy (1, 2).

We further investigated the presence and status of microglia/macrophages in the mouse model of OIR. In this experimental model of ischemia-driven NV, exposure of neonatal mice to hyperoxic stimulus leads to vaso-obliteration in the central retina. After returning to room air, this central retinal ischemia leads to the upregulation of proangiogenic factors and to pathological NV. The pathological NV reaches maximal severity at P17 (Figure 1E) (29). Using isolectin B4 (IB4) staining of retinal flat mounts, we found a dramatic activation of microglia, characterized by a hypertrophic amoeboid morphology, in OIR P17 retina. Those activated MPs were observed in central avascular area, midperipheral neovascular tufts, and peripheral normal vascular area (Figure 1F). In contrast, IBA1^+^ cells in the normal, control, room-air mouse retinas exhibited a ramified morphology. These results suggest both the activation of microglia/macrophages in human DR and a rodent model of ischemic retinopathy as well as the close association of MPs with neovessels in human PDR and mouse OIR.

### Long-acting GLP-1R agonist NLY01 ameliorates microglia activation and inhibits NF-κB p65 nuclear translocation in primary microglia.

With the prominence of the association of activated MPs with ischemic retina and neovessels in the mouse OIR model of ischemic retinopathy as well as in neovascular membranes in human PDR, we were interested in a strategy for therapeutic modulation of microglia. Thus, we examined NLY01, based on its previous efficacy in reaching the CNS and directly modulating microglia, thereby reducing neurodegeneration and behavioral deficits in a mouse model of Parkinson’s disease (18). In the present study, we further investigated the anti-inflammatory effect of NLY01 on microglia.

**Figure 2:**
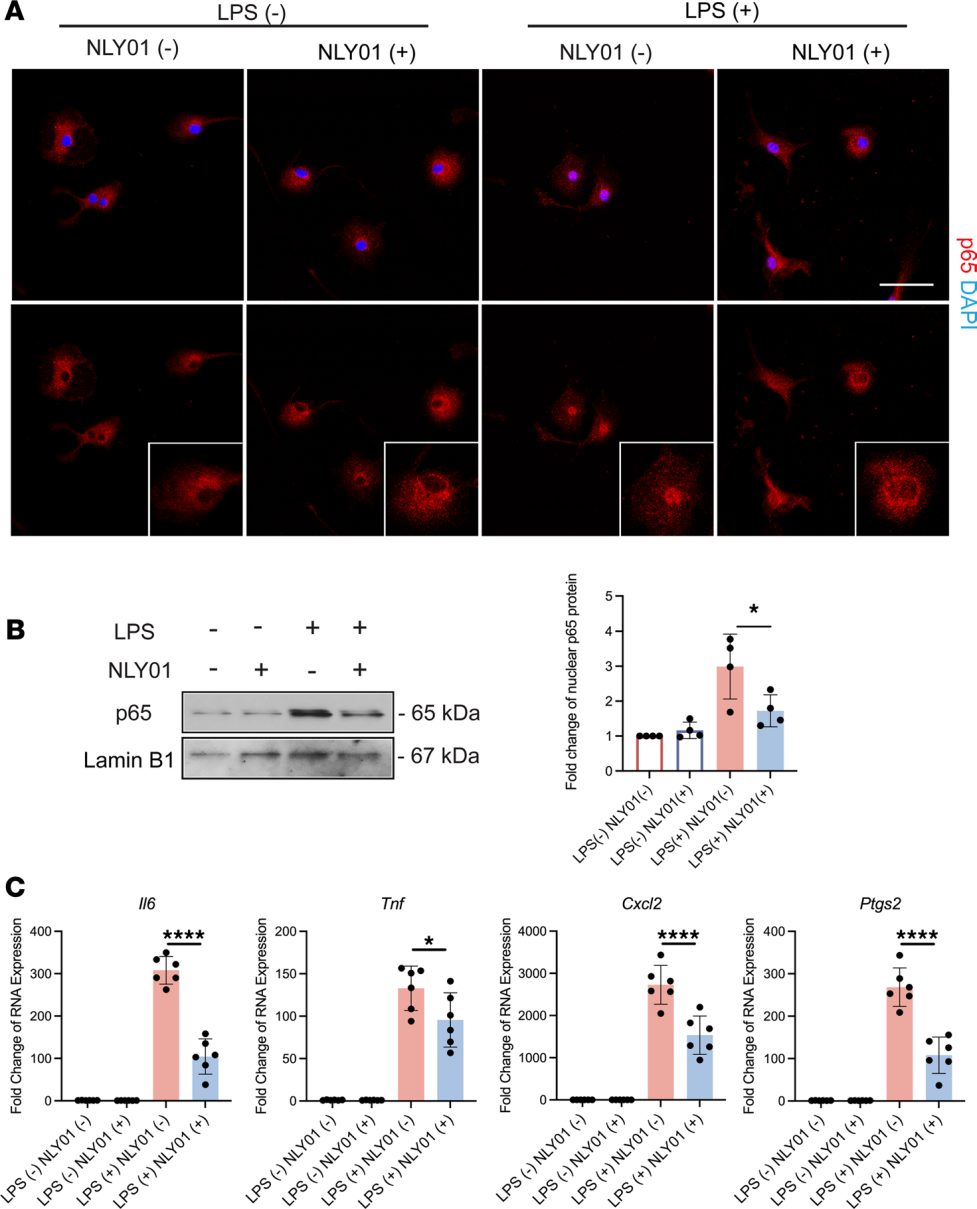
NLY01 regulates LPS-stimulated cultured microglia activation. (**A**) Representative images of NF-κB p65 (red) staining in LPS-stimulated primary brain microglia pretreated with vehicle or NLY01 (1 μM). Blue: DAPI staining. Scale bar: 50 μm. (**B**) Representative (top panel) and quantitation (bottom panel) Western blotting analysis of NF-κB p65 nuclear translocation in LPS-stimulated primary brain microglia with vehicle or NLY01 pretreatment. *P < 0.05. One-way ANOVA test was used for statistical analysis. Data are presented as mean ± SD (*n* = 4). The experiment was conducted 3 times with similar results. (**C**) Expression of inflammatory genes in LPS-stimulated BV2 cells with vehicle or NLY01 pretreatment. *P < 0.05. One-way ANOVA test was used for statistical analysis. Data are presented as mean ± SD (*n* = 6).

NF-κB is a pivotal proinflammatory transcription factor in microglia activation (30–32) that serves as a master regulator of multiple proinflammatory mediators. In cultured primary brain microglia, immunocytochemical analysis revealed dramatic activation of NF-κB by LPS, which was demonstrated by the nuclear translocation of p65. Pretreatment of microglia with NLY01 strongly suppressed LPS induction of NF-κB p65 nuclear translocation (Figure 2A). In parallel, we examined Western blot analysis of nuclear extracts of primary brain microglia. LPS significantly increased nuclear levels of p65. Pretreatment with NLY01 suppressed this increase in nuclear p65 in microglia (Figure 2B). We also examined inflammatory cytokine expression after NLY01 treatment in BV2 cells, a microglial cell line. As shown in Figure 2C, NLY01 pretreatment significantly reduced inflammatory cytokine expression induced by LPS stimulation. Together, these findings demonstrate direct modulation of microglial inflammatory activation by NLY01.

### NLY01 does not regulate the angiogenic phenotype of human retinal endothelial cells.

**Figure 3:**
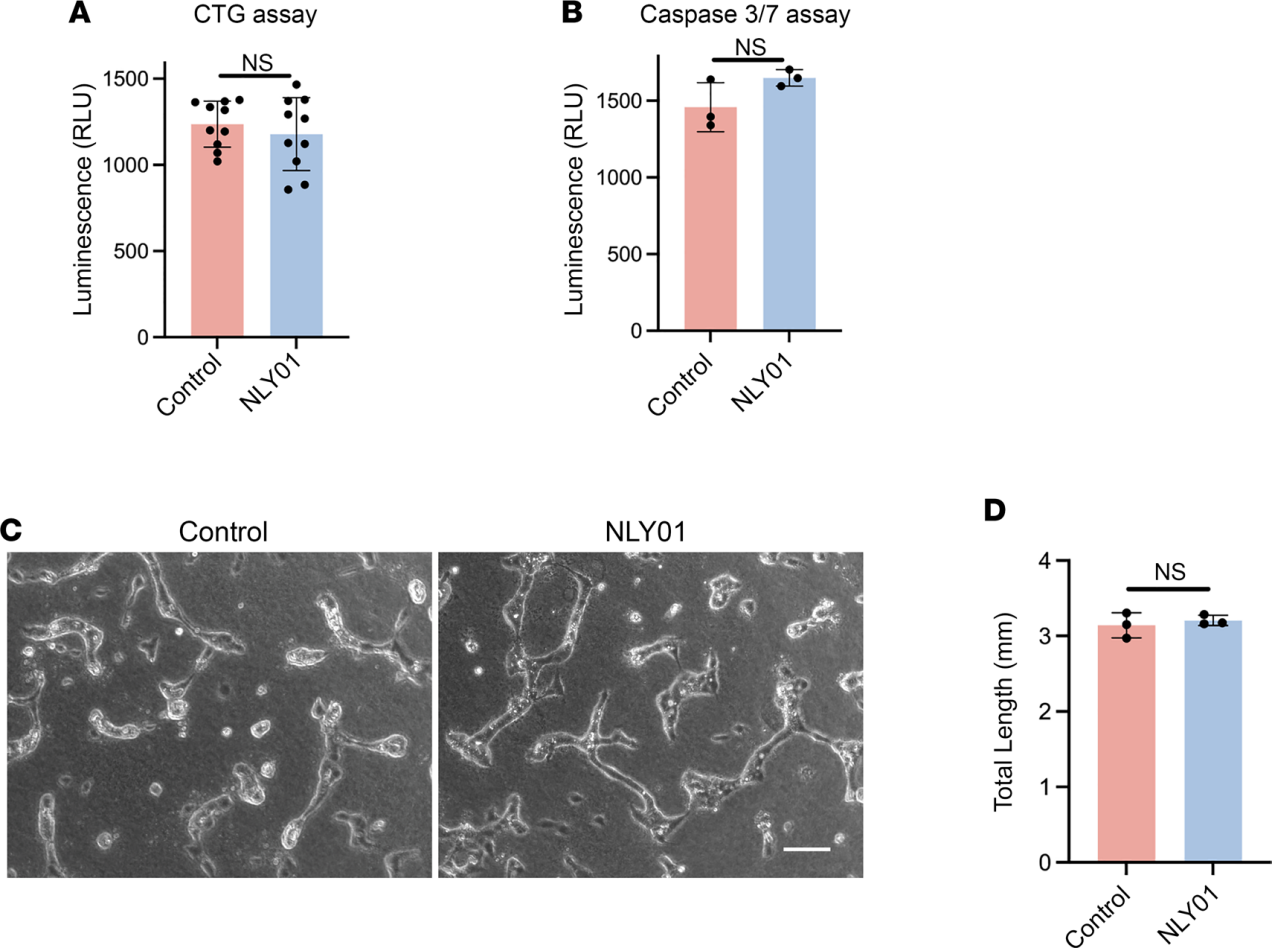
NLY01 does not exhibit a direct antiangiogenic effect on HRECs. HRECs were treated with NLY01 (1 μM) for 24 hours under hypoxic conditions. Cell viability and apoptosis were quantified by (**A**) CellTiter-Glo (CTG) luminescent cell viability assay (*n* = 10) and (**B**) Caspase 3/7 assay (*n* = 3), respectively. (**C**) Representative images and (**D**) quantitation of HREC tube formation with vehicle or NLY01 treatment (*n* = 3). An unpaired, 2-tailed Student’s *t* test was used for statistical analysis. Data are presented as mean ± SD. Scale bar: 100 μm.

Because our study focused on the effect of NLY01 on retinal angiogenesis, we looked for evidence of a direct effect of NLY01 on retinal endothelial cells. A previous study found no evidence of direct regulation of exenatide on barrier function of bovine retinal endothelial cells (23). Using primary human retinal endothelial cells (HRECs), we investigated possible direct effects of NLY01. We found no evidence of effect of NLY01 treatment on HREC viability (Figure 3A). Similarly, NLY01 treatment had no effect on HREC apoptosis, as demonstrated by caspase-3/7 activity (Figure 3B). We further investigated the effect of NLY01 on HREC angiogenesis, using the tube-formation assay, an in vitro correlate for blood vessel formation (33). We found no difference of endothelial tube length between NLY01- and vehicle-pretreated HRECs (Figure 3, C and D).

### NLY01 colocalizes with MPs in OIR retina.

We previously reported NLY01 modulation of microglia in the CNS with consequent neuroprotection in CNS models of neurodegeneration (18). Because of its modulation of inflammatory activation of microglia (Figure 3), we investigated NLY01 as a possible candidate modulator of microglial/macrophage activity in the retina.

**Figure 4:**
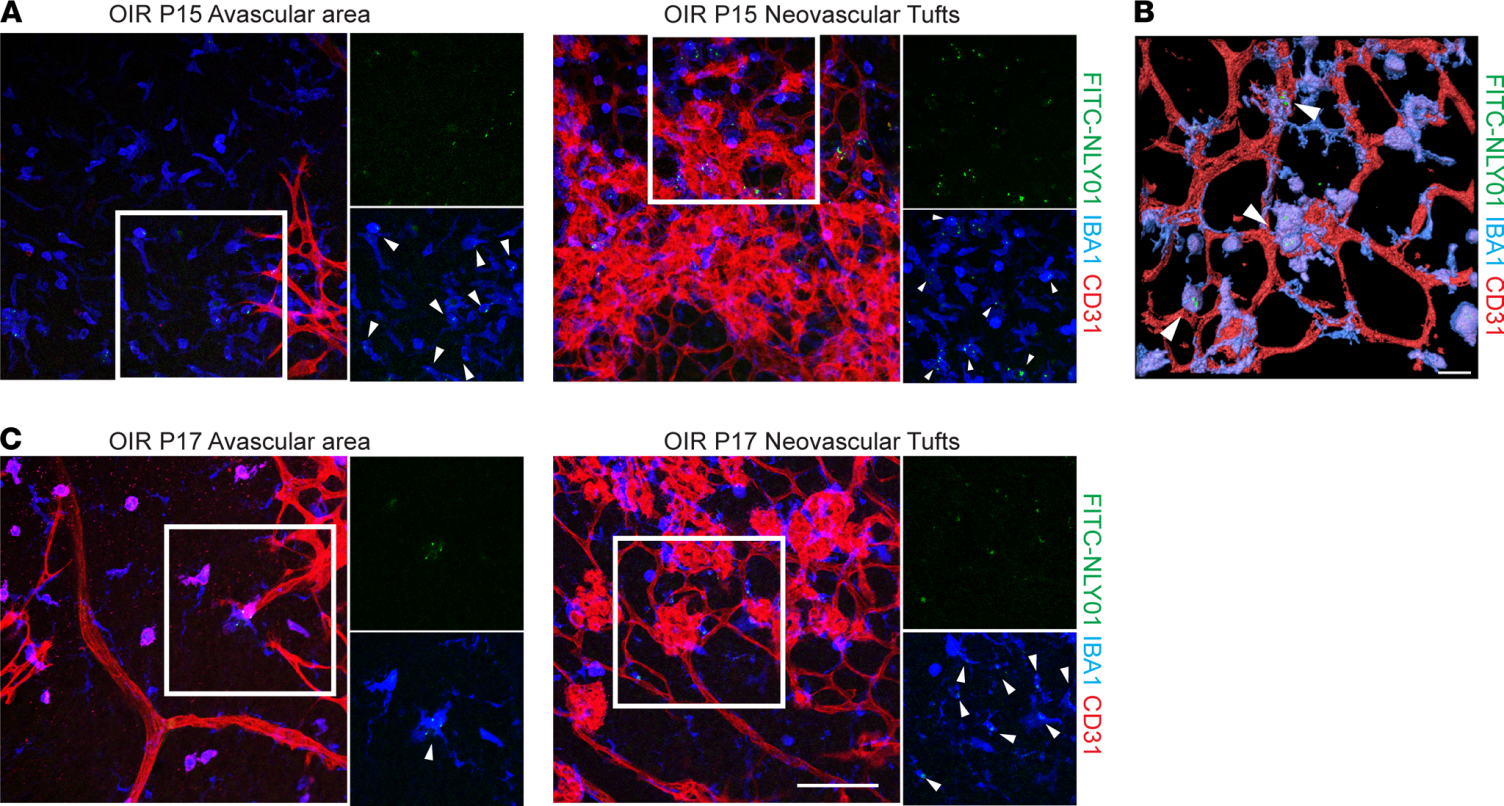
NLY01 colocalizes with MPs in OIR retina. FITC-NLY01 was injected intravitreally on P12. Representative images show the staining of FITC-NLY01 (green), NLY01 (blue), and CD31 (red). Enlarged images show FITC-NLY01 (green) colocalized with IBA1^+^ (blue) microglia/macrophages in both avascular areas and around neovascular tufts on (**A**) P15 and (**C**) P17 (*n* = 4). Scale bar: 100 μm. (**B**) 3D reconstruction image of FITC-NLY01 colocalization with MPs around the neovascular tufts in OIR retina. White arrowhead: FITC-NLY01 (green). Scale bar: 20 μm.

We first examined the distribution of NLY01 in retina following administration of FITC-tagged NLY01 at P12, visualizing its localization at P15 and P17. At P15, FITC-NLY01 was present in the avascular area and in association with neovascular tufts. There was clear colocalization with IBA1^+^ MPs (Figure 4A). With *Z*-stack and Imaris 3D (Bitplane AG) reconstruction, we further confirmed the colocalization of FITC-NLY01 with IBA1^+^ cells but not vascular endothelial cells (Figure 4B), suggesting that FITC-NLY01 was primarily localized to retinal microglia/macrophages. Five days after injection, FITC-NLY01 was still observed in P17 retina. Similar to P15, FITC-NLY01 primarily colocalized with IBA1^+^ cells in the avascular retina and neovascular tufts (Figure 4C).

### NLY01 modulates retinal microglia/macrophage numbers and activation in OIR retina.

**Figure 5:**
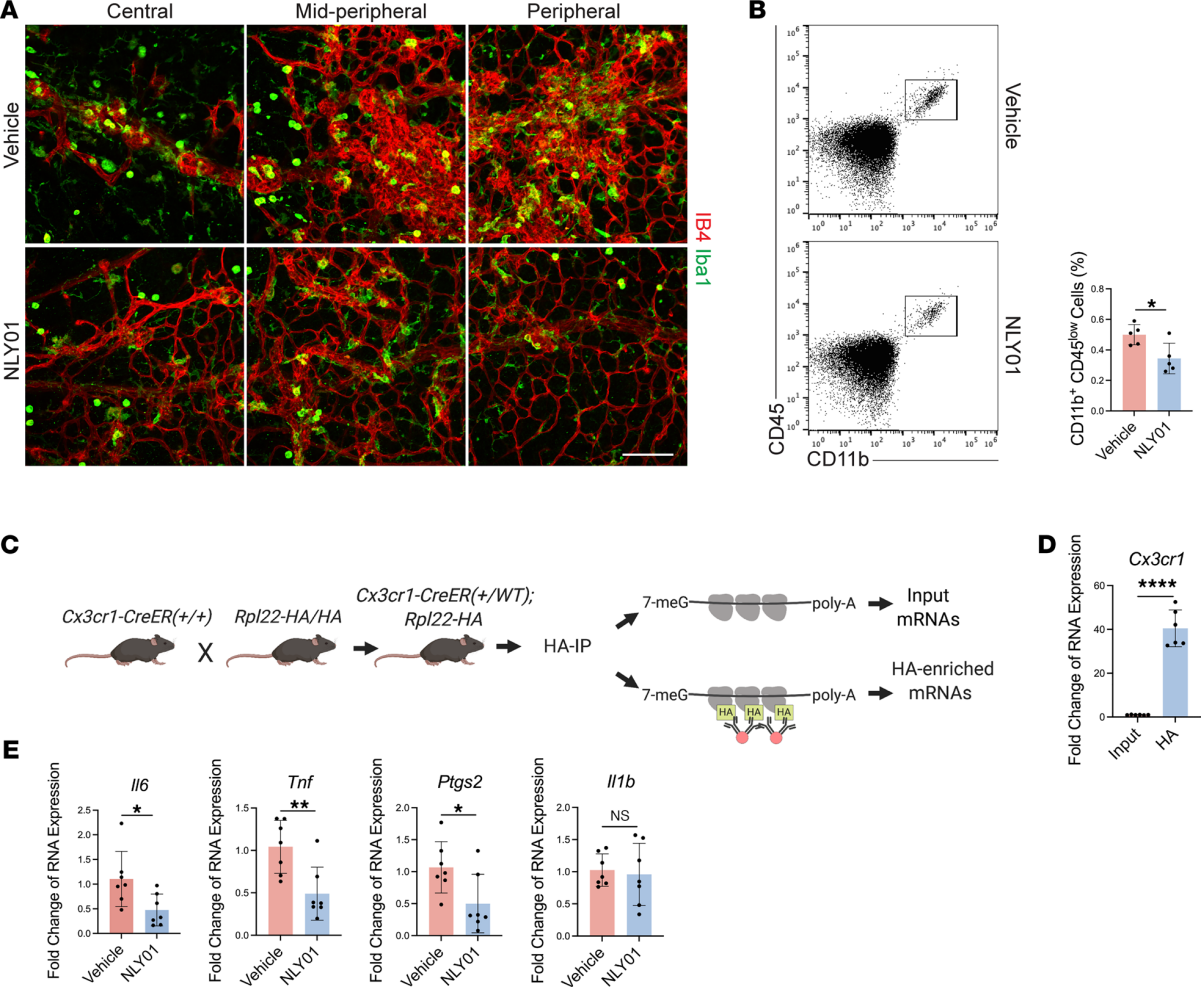
NLY01 modulates MP activation in OIR retina. (**A**) Representative images of vasculature (CD31, red) and microglia (IBA1, green) staining on retinal flat mounts from vehicle- or NLY01-treated eyes on P17 OIR (*n* = 3). Scale bar: 100 μm. (**B**) Representative flow cytometric plots and quantifications of CD11b^+^CD45^lo^ cells in P15 vehicle- and NLY01-treated retinas (*n* = 5). Each data point represents the value from 2 retinas. (**C**) Schematic diagram of RiboTag strategy. (**D**) Microglia/macrophage polyribosome RNA enrichment in the HA-pulldown samples. Input samples served as the control (*n* = 6). (**E**) Changes in inflammatory gene expression in MP polyribosome RNA enriched from vehicle- or NLY01-treated *Cx3cr1CreER:Rpl22HA* mice retinas (*n* = 7). Each data point represents the value for a single retina. *P < 0.05; **P < 0.01. An unpaired, 2-tailed Student’s *t* test was used for statistical analysis. Data are presented as mean ± SD.

Consistent with microglial activation in inflammatory CNS diseases, we observed a substantial increase in microglial/macrophage number and typical morphological changes in OIR (Figure 1). Given the current appreciation of OIR as a neuroinflammatory condition, this activation state of microglia could contribute to the retinal vascular pathology of OIR. Because NLY01 treatment modulated the neuroinflammatory phenotype of primary microglia (Figure 2), we investigated whether NLY01 could regulate myeloid activation in vivo in OIR retina. In retinal flat mounts, using IBA1 and IB4 staining, we observed a marked reduction in amoeboid-liked microglia/macrophages in the NLY01-treated eyes (Figure 5A). In addition, flow cytometric analysis demonstrated that the numbers of retinal microglia, represented by the CD11b^+^CD45^lo^ population (34), were significantly reduced in NLY01-treated eyes compared with vehicle-treated eyes at P15 (Figure 5B), suggesting that NLY01 treatment inhibits MPs in OIR retina. Individual flow cytometry plots are shown in Supplemental Figure 1.

We were also interested in whether NLY01 modulated microglial/macrophage production of critical cytokines that contribute to the proinflammatory milieu in OIR. To evaluate MP-specific gene expression in vivo, we utilized the RiboTag strategy for studying gene expression and mRNA translation in specific cell populations in vivo (35). In applying this RiboTag strategy for MPs, we retrieved the microglia/macrophage-specific translatomes from *Cx3cr1^CreER^:Rpl22HA* mice (Figure 5C) (36). This approach carries important advantages for microglia, including the exclusion of exogenous RNAs from microglial phagocytosis of surrounding cellular elements, allowing identification of mRNAs undergoing active translation in these cells. We first confirmed the enrichment of microglia/macrophage-specific translatomes by detecting MP-specific gene *Cx3cr1* expression in input and HA samples (Figure 5D). Retinal microglia/macrophages from NLY01-treated eyes expressed significantly lower levels of proinflammatory cytokines, including *Tnf*, *Il6*, and *Ptgs2*, than did the corresponding vehicle-treated OIR eyes (Figure 5E). Interestingly, we did not observe an NLY01 effect on expression of another proinflammatory cytokine, *Il1b*, in MPs. The NLY01 effect on *Tnf* is especially notable, given the previous direct implication of this cytokine on reparative and pathologic angiogenesis in OIR and the expression we observed in myeloid cells in human PDR membranes (1).

### NLY01 treatment reduces gene expression of inflammatory and angiogenic regulators in ischemic retina.

**Figure 6:**
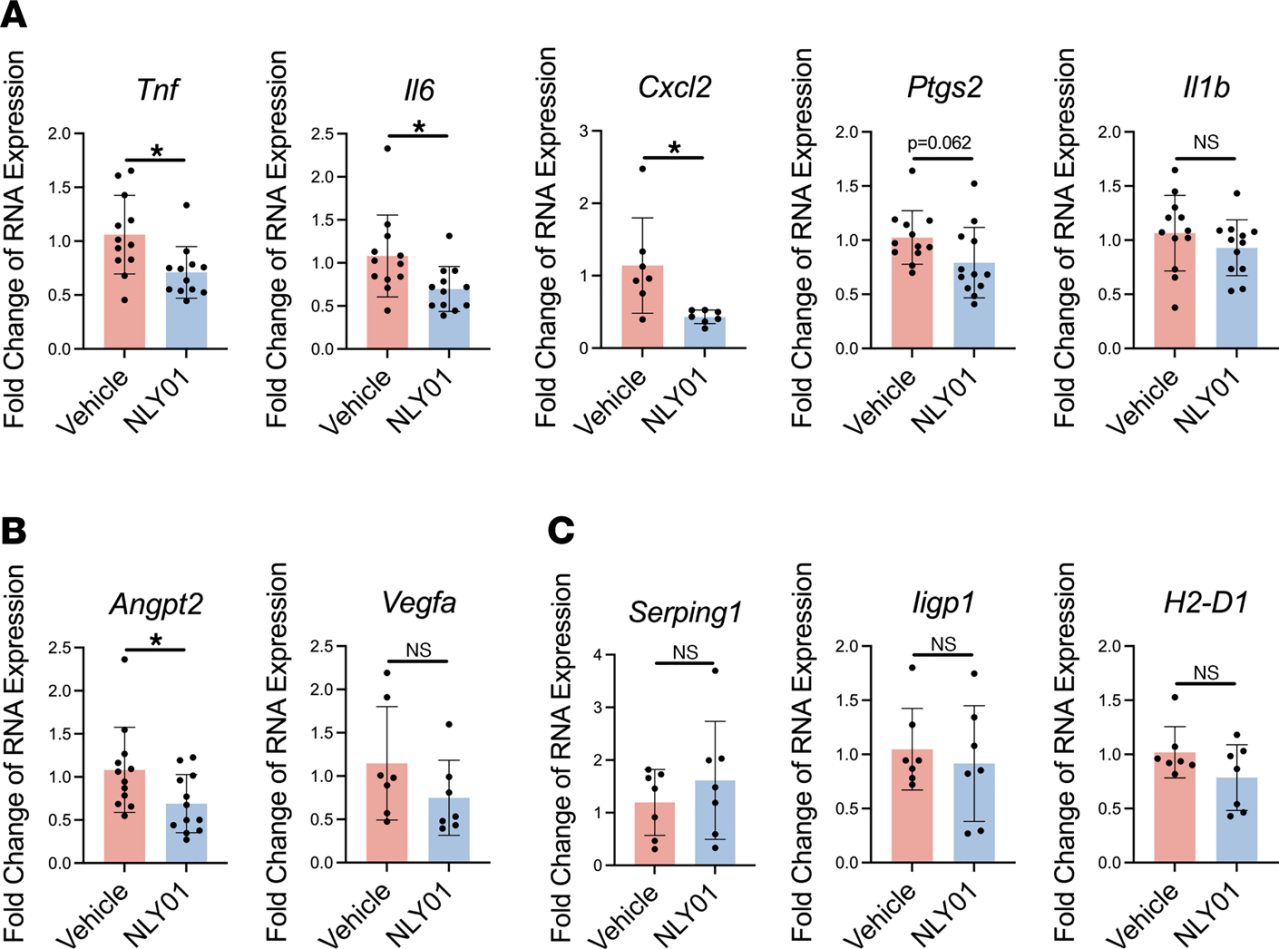
NLY01 reduces inflammatory and angiogenesis-related gene expression in OIR retina. (**A**) Inflammatory gene expression in vehicle- and NLY01-treated eyes. (**B**) Angiogenesis-related gene expression in vehicle- and NLY01-treated groups. (**C**) Gliosis-related gene expression (*n* = 7). Each data point represents the value for a single retina. *P < 0.05. Statistical analysis was done via unpaired, 2-tailed Student’s *t* test. Data are presented as mean ± SD.

We further evaluated whether NLY01 treatment regulates the level of proinflammatory cytokines in the whole retina in OIR. Compared with vehicle-injected eyes, mRNA levels of *Tnf*, *Il6*, and *Cxcl2* were significantly reduced in NLY01-treated eyes at P15 (Figure 6A). *Tnf* and *Il6* are classic inflammatory cytokines closely involved in the pathophysiology and progression of DR (37). *Cxcl2*, which is increased in the vitreous of patients with PDR, plays an important role in amplifying inflammation. We also examined the effect of NLY01 on expression of angiogenic regulators. NLY01-treated eyes exhibited significant reduction in retinal expression of *Angpt2*, the gene coding for angiopoietin 2 (Figure 6B), an important regulator of angiogenesis; levels of *Angpt2* are increased in hypoxia-treated cells and ischemic retina (38) as well as in eyes of patients with PDR (39). We did not observe any effect of NLY01 treatment on the expression of *Vegfa* (Figure 6B). In a previous study, researchers found that NLY01 reduces brain astrocyte activation as a consequence of microglial modulation (18). In the setting of OIR, however, we did not see any change in the expression of gliosis-related genes (Figure 6C).

### NLY01 enhances reparative angiogenesis and ameliorates pathological NV in the OIR model.

**Figure 7:**
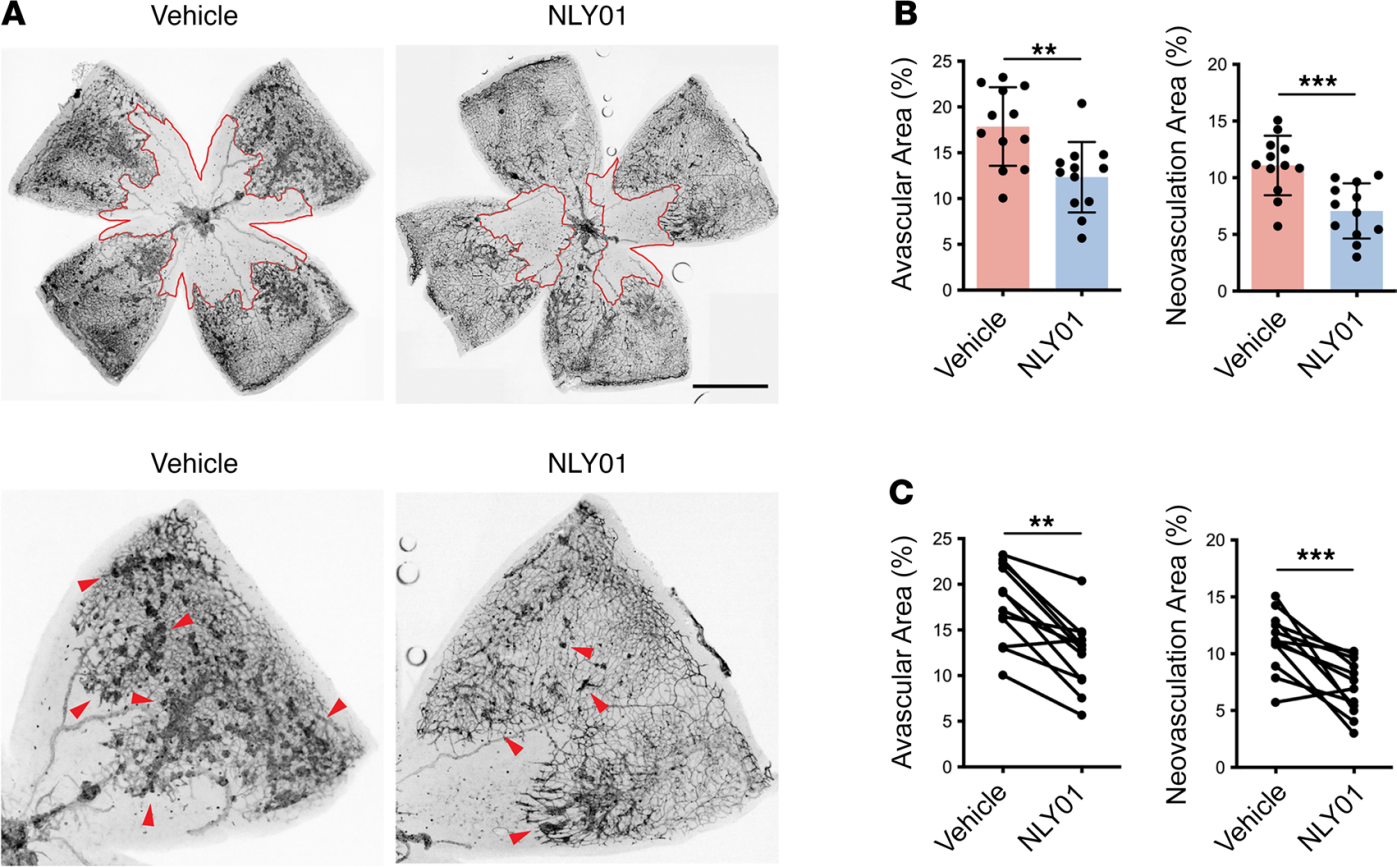
NLY01 treatment reduces avascular retinal area and neovascular tufts in OIR. (**A**) Representative retinal flat mounts from OIR P17 vehicle- or NLY01-treated retinas stained with IB4. (**B**) Quantitation of avascular area and NV tufts shown as a scatter plot–bar graph with unpaired analysis (*n* = 12). Each data point represents the value for a single mouse. **P < 0.01; ***P < 0.001. An unpaired, 2-tailed Student’s *t* test was used for statistical analysis. Data are presented as mean ± SD. (**C**) Quantitation of avascular area and NV tufts shown as a symbol-line graph with paired analysis (*n* = 12). Each data point represents the value for a single retina. **P < 0.01; ***P < 0.001. A paired, 2-tailed Student’s *t* test was used for statistical analysis. Scale bar: 1000 μm.

With its specific modulation of activated retinal microglia/macrophage with favorable effect on the inflammatory milieu, we examined whether NLY01 treatment at P12 regulates the retinal vascular response to ischemia in OIR. In this model, retinal vaso-obliteration is maximal at P9, and revascularization of the vaso-obliterated zone begins from P9 to P12 (29). Compared with vehicle-treated eyes, eyes treated with NLY01 at P12 had reduced avascular area at P17 (Figure 7, A and B), the peak of pathologic NV in OIR, indicating enhancement of reparative angiogenesis. Importantly, NLY01 treatment also resulted in significant reduction of pathologic retinal NV in the ischemic retina (Figure 7, A and B).

As an additional approach for analysis, we used paired statistical analysis to compare avascular retinal area and neovascular tufts between NLY01-treated eyes and their corresponding contralateral vehicle-treated eyes. This analysis also indicated significantly reduced avascular area and pathologic retinal NV area with NLY01 treatment as compared with vehicle (Figure 7C). These data indicate that NLY01 ameliorates vascular degeneration and pathological NV in the OIR model of ischemic retinopathy.

## Discussion

Although there has long been speculation that microglia play an important role in regulating the vasculature in ischemic retinopathies, their actual effects in retinal ischemia have remained understudied. In this study, we found evidence for activation of microglia in ischemic retina in humans and mice. We also found a strong colocalization of microglia/macrophages in intimate association with neovessels in human PDR specimens. Similarly, activated MPs colocalized around neovessels in the mouse OIR model, with a higher density relative to the physiologic vessels.

With multiple previous studies demonstrating GLP-1R agonists’ therapeutic modulation of microglia, particularly in CNS disease models, we selected a pegylated, long-acting exenatide, NLY01, which directly regulates activated microglia with favorable downstream effect on neurodegeneration, both in the α-synuclein preformed fibril mouse model of Parkinson’s disease as well as in the human A53T α-synuclein transgenic mouse model of α-synucleinopathy–induced neurodegeneration (18).

In the present study, we found that NLY01 suppressed inflammatory activation of cultured primary brain microglia cells. In contrast, we did not find evidence of a direct effect of NLY01 on cultured retinal endothelial cells with respect to viability, apoptosis, or endothelial cell tube formation. This is consistent with findings from a previous study in which there was no effect of exenatide on barrier function of cultured HRECs (23). Using FITC-labeled NLY01, we found that administration of NLY01 resulted in retinal bioavailability in the OIR model, with selective colocalization with retina MPs. These effects of NLY01 were associated with modulation of the neuroinflammatory phenotype of microglia/macrophages in vivo. Finally, along with microglial modulation, NLY01 significantly reduced retinal ischemia and pathologic retinal NV in OIR. Together, these findings suggest a significant role for microglia/macrophages in regulating the retinal vasculature in a disease setting, specifically with regard to angiogenesis, indicating that therapies targeting MPs could be a new approach for treating ischemic retinopathy and retinal neovascular disease.

There has been increasing evidence supporting a role for microglia in regulating ophthalmic and retinal disease entities. Microglia have become the focus of attention with regard to their active role in photoreceptor degeneration. Microglial phagocytosis of living rods accelerates photoreceptor death in inherited retinal degenerations (13). On the other hand, in the same context of inherited retinal degenerations, complement-mediated microglial phagocytic clearance of apoptotic rod photoreceptors can play a protective homeostatic role that limits cell death of surrounding healthy photoreceptors (27). In a light-damage model of photoreceptor degeneration, microglia migrated to the subretinal space and promoted the structural integrity of the retinal pigment epithelium (40). Collectively, these studies suggest that optimal modulation of microglia is warranted to provide the best clinical outcome in photoreceptor degenerative disorders.

Beyond primary photoreceptor degenerative diseases, microglia have recently been implicated in the initiation of retinal neuroinflammation in an experimental model of autoimmune uveitis (41). In the present study, we extend the scope of microglial function in retinal disease and suggest it also has a direct role in regulating the fate of pathologic neovessels in retinal ischemia. An important challenge in research on microglia and CNS studies is the difficulty in differentiating microglia from monocyte-derived macrophages, due to lack of markers for definitive distinction of these cell types (34). In addition, microglia and macrophages could potentially exhibit changes in markers such as CD45, which have been used to distinguish these cell types. For instance, in certain disease settings, microglia exhibit upregulation of *CD45* expression. In our study, it is probable that modulation of resident retinal microglia is a major, and possibly predominant, mechanism for NLY01 effect. This suggestion is based both on findings from a recent study demonstrating that microglia are the dominant myeloid cell in OIR (15) and the findings from our study showing that NLY01 treatment in OIR downregulated levels of CD11b^+^CD45^lo^ cells, which are typically acknowledged to be microglia (34). However, under certain conditions, infiltrating macrophages can assume a microglial phenotype (42). For this reason, in this study, we broadened our interpretation to include the possibility that the MPs observed in ischemic retina and modulated by NLY01 could represent monocyte-derived macrophages as well as microglia.

The OIR model has been intensively studied as a model of ischemia-induced NV (29, 43). This model has yielded tremendous insights, including the implication of VEGF as a major promoter of retinal angiogenesis (44), which strongly contributed to the development of anti-VEGF therapies that are now in widespread clinical use. Apart from proangiogenic growth factors, the role of neuroinflammation is well established in this model. For instance, investigators have suggested there is an important role for the proinflammatory, retinoic acid–related, orphan receptor-α in its stimulation of retinal NV in OIR via direct transcriptional control of suppressor of cytokine signaling 3. In addition, genetic deletion of *TNF-α* resulted in marked improvement of reparative angiogenesis and reduction of pathologic retinal NV in OIR (1). In parallel, the levels of multiple proinflammatory cytokines, including TNF-α and IL1- β, are increased in the vitreous of patients with PDR (37, 45) and retinopathy of prematurity (46), further supporting the importance of inflammation in driving ischemia-induced NV. As the dominant immune cell type in the retina in OIR (15), microglia likely play a major role in the inflammatory retinal milieu. Our studies indicate the NLY01 treatment inhibited microglial inflammatory activation in vitro and suppressed microglial activation in OIR in vivo. This was associated with both suppression of microglia-specific expression of cytokines as well as overall retinal levels of cytokines, including *Tnf*. Our findings further highlight the potential importance of TNF-α, especially in light of the co-expression of this cytokine with IBA1+ cells in human PDR (Figure 1D). Although NLY01 treatment reduced retinal expression of *Angpt2*, it had no effect on *Vegfa* expression. In this regard, pharmacologic modulation of microglia likely represents a VEGF-independent mechanism for treating ischemia-induced retinal NV.

The role of nonvascular elements, such as neurons and Müller glia, in regulating retinal NV is well appreciated. In recent years, great strides have been made in the investigation of myeloid cells and microglia, and the question of the nature of their influence on retinal NV has been raised. A subset of myeloid cells that express neuropilin-1 was found to regulate pathologic retinal NV in OIR via the vasorepulsive semaphorin, Sema3A. These proangiogenic myeloid cells were identified as MPs: either blood-derived macrophages and/or resident microglia (9). Strikingly, this same NRP-1^+^ myeloid cell population was found to be dispensable for developmental retinal angiogenesis (14). A recent study demonstrated a dramatic increase in the numbers of IBA1^+^ retinal myeloid cells in both the central avascular and peripheral vascularized area in OIR, indicating both myeloid cell proliferation and migration. There was a particular concentration of these myeloid cells, which were confirmed to be microglia, in the pathologic retinal neovessels (15). From a clinical context, investigators have demonstrated a significant increase in both the number and activation status of microglia around the native retinal blood vessels in the eyes of patients with NPDR (24). In the present study, we found prominent localization of microglia/macrophages with retinal neovessels in neovascular membranes of patients with PDR. Whether the vascular localization and activation of microglia represent a reactive response of microglia or an active role of these cells is an active topic of inquiry. Our present study adds to the present literature by demonstrating an active role for microglia, broadening the neurovascular framework of nonvascular cells that influence the retinal vasculature in disease.

With their high efficacy for glycemic control, GLP-1R agonists, including exenatide, are in common clinical use for patients with diabetes (47). More recently, the potential utility of these drugs has expanded to nondiabetic contexts, especially with respect to neurodegenerative diseases of the CNS, including Alzheimer’s and Parkinson’s diseases (48). An important facet of this benefit is the modulation of microglia. In these and other chronic diseases, long-acting formulations of GLP-1R agonists could be of tremendous benefit. Notably, NLY01 has demonstrated strong benefit via direct modulation of CNS microglia in a mouse model of Parkinson’s disease, with good delivery of the drug to the brain (18). These findings have driven interest in the possibility of repurposing these existing GLP-1R agonists, including exenatide, for nondiabetic conditions (49). Indeed, a randomized, placebo-controlled clinical trial has demonstrated therapeutic benefit of exenatide in Parkinson’s disease (19). The bioavailability of this and other GLP1 analogues in the CNS and retina, as well as the development of long-acting formulations for chronic disease conditions, will be of great interest. It is worth mentioning that multiple studies have reported that GLP-1R agonists, including exendin-4 (exenatide), can exert their beneficial role through mechanisms independent of GLP-1R (50, 51), and further definitive elucidation of effects on microglia will be of great interest.

In light of their already beneficial utility in the management of diabetes, the use of NLY01 and other GLP-1R agonists is particularly promising in their potential for the treatment of PDR, the leading cause of blindness in working-age adults in developed countries worldwide. Our study supports the importance of microglia as an important cellular regulator in ischemic retina and raises the possibility of this pharmacologic strategy for microglial modulation for the treatment of retinal neovascular disorders, including PDR.

## Methods

### Human sample collection.

Human donor eyes were obtained and processed as previously described (52). All eyes were fixed in 10% formalin for at least 24 hours within 18 hours postmortem. PDR membranes were collected from patients undergoing vitreoretinal surgery and fixed in 10% formalin as previously described (53).

### Preparation and characterization of NLY01.

NLY01 is a pegylated (Cys40) exendin-4 with the amino acid sequence His-Gly-Glu-Gly-Thr-Phe-Thr-Ser-Asp-Leu-Ser-Lys-Gln-Met-Glu-Glu-Glu-Ala-Val-Arg-Leu-Phe-Ile-Glu-Trp-Leu-Lys-Asn-Gly-Gly-Pro-Ser-Ser-Gly-Ala-Pro-Pro-Pro-Ser-Cys(PEG50K)-NH2. NLY01 was synthesized and characterized as described (18, 54, 55) and provided by Neuraly Inc.

### IHC.

We performed IHC as previous described (53, 56). Deparaffinized sections were boiled in 1× target retrieval solution (Dako; Agilent Technologies) for 20 minutes. After blocking in 5% normal goat serum diluted in PBS, the sections were incubated with anti-IBA1 antibody (Wako) or the isotype- and concentration-matched normal IgG control overnight at 4°C. After 3 washes with PBS plus 0.1% Tween 20, sections were then incubated with the secondary biotinylated goat anti-rabbit IgG for 1 hour at room temperature. Sections were then detected by the alkaline phosphatase detection system (Vectastain ABC-AP kit; Vector Laboratories), and a blue reaction product was produced by incubating sections with alkaline substrate (Vector blue AP substrate kit III; Vector Laboratories). Sections were also stained with H&E. For immunofluorescence staining, samples were blocked with 5% normal donkey serum diluted in TBS with 0.1% Triton X-100 (TBST) for 2 hours after boiling in target retrieval solution. The samples were incubated with anti-IBA1 antibody (Wako) and anti–TNF-α antibody (R&D Systems) overnight at 4°C. After 3 washes with TBST, samples were incubated with Alexa Fluor 594 donkey anti-rabbit antibody and Alexa Fluor 488 donkey anti-goat antibody (Thermo Fisher Scientific). Photographs were taken using a Zeiss LSM 880 with a ×20 objective lens. Antibodies used for staining are listed in Supplemental Table 1 (supplemental material available online with this article; https://doi.org/10.1172/jci.insight.93382DS1).

### Animals.

C57BL/6J mice, *CX3CR1YFP-CreER/YFP-CreER* (catalog 021160) mice and *RiboTag* (*B6J.129(Cg)-Rpl22tm1.1Psam/SjJ*; catalog 029977) mice were purchased from the Jackson Laboratory. The animals were maintained on an AIN-76A diet and water ad libitum and housed at a temperature range of 20°C–23°C under 12:12 hour light/dark cycles.

### Mouse model of OIR and NLY01 treatment.

The mouse OIR model was described previously (29). Pups were placed in 75% oxygen chamber with their nursery mother for 5 days from P7 to P12. At P12, mice were removed from the chamber and returned to room air. For the NLY01 treatment, pups received an intravitreal injection on P12 of NLY01 (5 μg/μL) or NLY01-FITC in 1 eye and PBS in the contralateral eye as control. Mice were euthanized) at different time points for additional experiments.

### Retinal whole-mount preparations and staining.

Retinal whole-mount staining was performed as previously described (57). Briefly, mice were euthanized on P17. Mice with body weight less than 6 g on P17 were excluded from analysis. Whole eyes were fixed in 4% paraformaldehyde for 30 minutes and the retinas were carefully dissected. After blocking with 10% normal goat serum in PBS plus 0.3% Triton X-100 (PBST), the retinal vessels were visualized by incubating overnight with Alexa Fluor 594–conjugated isolectin GS-IB4 from *Griffonia simplicifolia* (Life Technologies). For the microglia and endothelial cell staining, retinas were incubated with rabbit anti-mouse IBA1 antibody and rat anti-mouse CD31 antibody (BD Biosciences) at 4°C overnight. The retinas were then extensively washed in PBST for 3 hours with changes of PBST every 30 minutes, followed by labeling with goat anti-rabbit IgG conjugated with Alexa Fluor 647 antibody (Invitrogen) and goat anti-rat IgG conjugated with Alexa Flour 594 at 4°C overnight. After washing with PBST, retinas were flat mounted on slides in Fluoromount-G (Electron Microscopy Sciences). Photographs were taken using a Zeiss LSM 710 with a ×20 objective lens. Imaris software (version 8.1.2) was used for 3D reconstructions of the confocal Z-stacks. Antibodies used for staining are listed in Supplemental Table 1.

### Quantitation of retinal vaso-obliteration and pathologic NV.

Retinal vaso-obliteration and NV were quantitated as previously described (58). Briefly, images of each of the 4 quadrants of retina were imported into Adobe Photoshop. Retinal segments were merged to produce 1 image of each entire retina. Vaso-obliteration and pathologic NV areas were quantified by comparing the number of pixels in the VO or NV area with the total number of pixels in the whole retina area.

### Real-time PCR analysis.

RNA was isolated using the RNeasy mini kit (QIAGEN) and single-stranded cDNA was synthesized using M-MLV Reverse Transcriptase (Invitrogen) as previously described (59). Quantitative PCR was performed with SYBR Green PCR Master Mix (Invitrogen) with the StepOnePlus real-time PCR system (Applied Biosystems). The quantitative PCR primers are described in Supplemental Table 2. *Ppia* was used for normalization.

### Retinal flow cytometry.

Retinal flow cytometry was performed as previously described (60). Retinas were collected on P15 or P17 and then digested with papain (1.65 U/mL; Worthington Biochemical) by incubation in a 37°C water bath for 16 minutes with a gentle swirl of the tube every 8 minutes. Tissues were then disaggregated by gentle pipetting up and down, and dropwise passing back into the tube through a serological pipette in low-ovomucoid solution. Single-cell suspensions of tissues were obtained by passing through a 70 mm cell strainer (BD Biosciences). The cells were then washed twice with PBS containing 5% FBS, 0.05% NaN_3_, and HEPES (10 mM) and blocked with rat anti-mouse CD16/32 antibody (BD Biosciences) for 5 minutes at 4°C. Cells were incubated with rat anti-mouse APC-CD11b (eBioscience) and FITC-CD45 (eBioscience) for 30 minutes at room temperature. Cells were washed 3 times. A 4-laser Sony SH800 cell sorter (Sony Biotechnology) was used to collect the data, and FlowJo software was used for analysis. Antibodies used for staining are listed in Supplemental Table 1.

### Ribosome IP.

Isolation of polysome-bound mRNA using RiboTag from tissues was performed as previously described (36). Retinas were homogenized in 400 μL of polysome buffer (50 mM Tris, pH 7.5, 100 mM KCl, 12 mM MgCl_2_, 1% NP-40, 1 mM DTT, 200 U/mL RNasin, 1 mg/mL heparin, 100 μg/mL cycloheximide, and 1× protease inhibitor) using pellet pestles (Kimble Chase) on ice. The homogenate was centrifuged at 15,300*g* for 10 minutes at 4^o^C, and the supernatant was collected for input. The polysome-bound mRNA was isolated with the IP with HA antibody. Briefly, 25 μL of anti-HA antibody-conjugated magnetic beads (M180-11; MBL International) was added to the retinal supernatant and incubated at 4°C overnight on an orbital shaker. The next day, the beads were washed 4 times with high-salt buffer (50 mM Tris, pH7.5, 300 mM KCl, 12 mM MgCl_2_, 1% NP-40, 1 mM DTT, and 100 μg/mL cycloheximide). Beads were then resuspended in 350 μL RLT buffer plus β-mercaptoethanol, thoroughly vortexed, and pelleted using a magnetic separator. The supernatant was transferred into a new tube and RNA was extracted using RNeasy Micro kit (QIAGEN), according to manufacturer’s instructions.

### Primary microglia isolation.

Primary microglia were isolated from brain as previously reported (18). Briefly, brain tissues from C57 mice at P1 were obtained and washed 3 times with complete medium (DMEM/F12 supplemented with 10% heat-inactivated FBS, 50 U/mL penicillin, 50 μg/mL streptomycin, 2 mM l-glutamine, and 100 μM nonessential amino acids with 2 mM sodium pyruvate). Brain tissues were then digested with 0.25% trypsin-EDTA at 37°C for 10 minutes and washed 3 times with complete medium. Single-cell suspension then was obtained by trituration. Cell debris and aggregates were removed by passing the single-cell suspension through a 70 μm nylon mesh. The final single-cell suspension was cultured in T75 flasks for 13 days, with a complete medium change performed on day 6. Microglia were isolated using the EasyStep Mouse CD11b Positive Selection Kit (StemCell).

### Cell culture and treatment.

Primary microglia were cultured with incomplete medium (DMEM/F12 supplemented with 50 U/mL penicillin, 50 μg/mL streptomycin, 2 mM l-glutamine, and 100 μM nonessential amino acids with 2 mM sodium pyruvate) overnight. BV2 cells were provided by Kannan Rangaramanujam from the Center for Nanomedicine, Department of Ophthalmology, Wilmer Eye Institute, Johns Hopkins University. Cells were pretreated with NLY01 (1 μM) for 1 hour followed by LPS treatment at a concentration of 100 ng/mL for 30 minutes. HRECs (Cell Systems) were cultured in EGM2-MV medium (Lonza) in a humidified 5% CO_2_ incubator at 37°C, and medium was changed every 2–3 days. HRECs were grown in fibronectin-coated (Invitrogen) dishes and were used at passages 6–10. For NLY01 treatment, cells were cultured in EGM2 without FBS (Invitrogen) overnight and then treated with NLY01 (1 μM) overnight in a humidified incubator.

### Immunofluorescence staining of NF-κB in primary microglia.

Cells were seeded in a 35 mm dish at the density of 10^5^ cells/well. After treatment, cells were fixed with 4% paraformaldehyde for 20 minutes at room temperature. After being washed with PBS, cells were permeabilized with 0.5% Triton X-100 in PBS for 5 minutes. Cells were then blocked with 4% BSA in TBS with 0.05% Tween for 60 minutes at room temperature. Cells were incubated with rabbit anti-mouse NF-κB p65 (Cell Signaling Technology) and goat anti-mouse IBA1 (1:100; Novus Biologicals) for 60 minutes at room temperature. After being washed 3 time with TBS with 0.05% Tween, cells were then incubated with donkey anti-rabbit IgG conjugated with Alexa Flour 594 (Invitrogen) and donkey anti-goat IgG conjugated with Alexa Flour 647 (Invitrogen). Cells were washed 3 times with TBS with 0.05% Tween, followed by DAPI staining. Photographs were taken using a Zeiss LSM 710 with a ×40 objective lens.

### Nuclear extract preparation and Western blot analysis.

Nuclear extracts from primary microglia were prepared using NE-PER Nuclear and Cytoplasmic Extraction Reagents (Thermo Fisher Scientific) as previous described (61). Proteins were quantified by the BCA protein assay (Bio-Rad) and protein extracts were aliquoted and stored at −80°C. Rabbit anti-mouse NF-κB p65 antibody (Cell Signaling Technology) was used at a 1:1000 dilution. Lamin B antibody (1:400; Santa Cruz Biotechnology) and histone A3 antibody (1:2000; Cell Signaling Technology) were used for loading control normalization. After incubating with primary and secondary antibodies, the blots were detected with femto chemiluminescent substrates (Thermo Fisher Scientific).

### Cell proliferation assay and apoptosis assay.

HREC proliferation activity was measured using the CellTiter-Glo Luminescent Cell Viability Assay (Promega Corp.) following the manufacturer’s instruction. For the measurement of in vitro apoptosis, caspase-3/7 activity was measured using a Caspase-Glo 3/7 assay kit (Promega Corp.) according to the manufacturer’s instructions. After incubation, the plates were shaken thoroughly, and absorbance was measured using a microplate reader (FLUOstar OPTIMA; BMG Labtech GmbH) at 492 and 620 nm.

### Tube-formation assay.

HREC tube formation was evaluated using a 2-layer collagen gel mixture assay, as previously described (62). HRECs were trypsinized, and 7 × 10^4^ cells were seeded on top of the lower collagen gel layer in each well of the 24-well plate. After overnight culture, cells were then treated with NLY01 in EBM2 plus 2% FBS for 5 hours. Medium was then removed, and 150 μL collagen gel mixture was added to each well. After 2 hours of gel polymerization at 37°C, 500 μL of medium containing NLY01 was added to the upper layer of collagen gel. Eighteen hours later, 6 random fields in each well were chosen and photographed using an Axiovert 200 M microscope (Carl Zeiss Microscopy).

### Statistics.

Data were presented as mean ± SD. Unpaired, 2-tailed Student’s *t* test was used to perform statistical analysis between 2 groups. One-way ANOVA was used to perform statistical analysis between multiple groups. There was no intentional removal of any outcome measures. Analysis was performed using GraphPad Prism, version 8. *P* < 0.05 was considered statistically significant.

### Study approval.

Human donor eyes were obtained from the Wilmer Eye Institute Ophthalmic Pathology laboratory with Johns Hopkins Medicine IRB approval. All the animal procedures were approved by the Institutional Animal Care and Use Committee of the Johns Hopkins University School of Medicine and were conducted in accordance with the Association for Research in Vision and Ophthalmology Statement for the Use of Animals in Ophthalmic and Visual Research.

## Author contributions

LZ was responsible for the experimental design; performed intravitreal injection experiments, immunostaining, flow cytometry, and cell culture studies; and wrote and edited the manuscript. ZX was responsible for the experimental design and cell culture studies and edited the manuscript. YO was responsible for generating the NLY01 and critical discussion of experiment design. RG was responsible for ribosome IP. GL and HC contributed to the mouse experiments. YX participated in experiments with primary microglia. SL provided critical discussion of results and reviewed and edited the manuscript. EJD contributed to the experimental design and analysis of data, wrote and edited the manuscript, and is the guarantor of this work. All authors approved the final version of manuscript to be published.

## Supplementary Material

Supplemental data

## Version 1 10/21/2021

In-Press Preview

## Version 2 12/08/2021

Electronic publication
