# Supplementary material for: Myeloid cell modulation by a GLP-1 receptor agonist regulates retinal angiogenesis in ischemic retinopathy
Source: JCI Insight. 2021 Dec 8;6(23):e93382. doi: 10.1172/jci.insight.93382 (PMC8675187; doi:10.1172/jci.insight.93382)
Supplement: Supplemental data [file jciinsight-6-93382-s127.pdf]

Supplemental figure 1

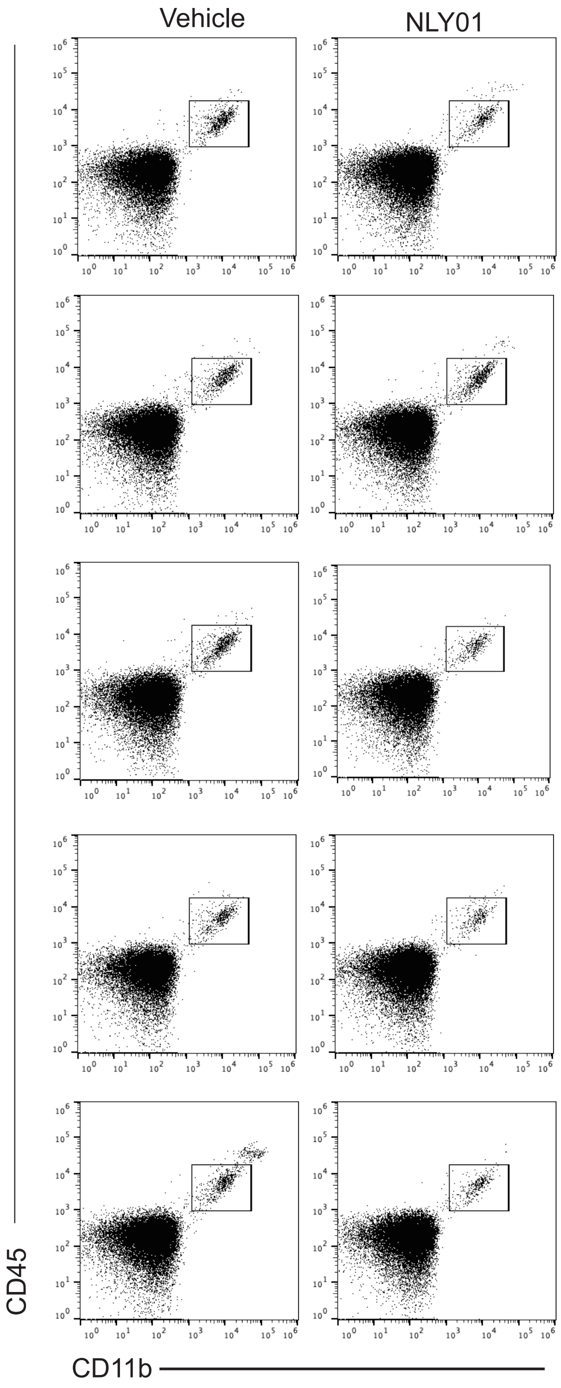

S1. Flow cytometric analysis of retinas following NLY01 treatment. Flow cytometric plots of CD11b+ CD45low cells in P15 vehicle- and NLY01-treated retinas. Left panels are the retinas from vehicle-treated eyes. The right panels show contralateral retinas from NLY01-treated eyes from the same mice. n=5. Each sample consisted of two retinas pooled together for flow cytometric analysis.

Supplementary Material

Table 1. Antibodies used for immunostaining, FACS and Western blot

Antibody	Source	Identifier	Dilution
Anti Iba1, Rabbit (for Immunocytochemistry)	FUJIFILM Wako	019-19741	1:100
Isolectin GS-IB4, Alexa Fluor™ 594 Conjugate	Invitrogen	I21413	1:200
Purified Rat anti-Mouse CD16/32	BD Bioscience	553142	1:100
CD45 Monoclonal Antibody (30-F11), FITC	Invitrogen	11-0451-82	1:100
CD11b Monoclonal Antibody (M1/70), APC	Invitrogen	17-0112-82	1:100
AIF-1/Iba1 Antibody	Novus	NB100-1028	1:100
NF-κB p65 (D14E12) XP® Rabbit mAb	Cell Signaling	8242S	1:100 (IF)<br>1:1000(WB)
Human/Mouse TNF-alpha antibody	R&D	AF-410	1:200
Histone H3 (D1H2) Rabbit mAb	Cell Signaling	4499	1:2000
Goat anti-Rabbit (H+L) Cross-Adsorbed Secondary Antibody, Alexa Fluor 647	Invitrogen	A-21244	1:500
Goat anti-Rat (H+L) Cross-Adsorbed Secondary Antibody, Alexa Fluor 594	Invitrogen	A-11007	1:500
Goat anti-Rabbit IgG (H+L) Cross-Adsorbed Secondary Antibody, Alexa Fluor 488	Invitrogen	A-11008	1:500
Donkey anti-Goat IgG (H+L) Highly Cross-Adsorbed Secondary Antibody, Alexa Fluor Plus 488	Invitrogen	A-32814	1:500
Donkey anti-Rabbit IgG (H+L) Highly Cross-Adsorbed Secondary Antibody, Alexa Fluor 594	Invitrogen	A-21207	1:500
Donkey anti-Goat IgG (H+L) Highly Cross-Adsorbed Secondary Antibody, Alexa Fluor Plus 647	Invitrogen	A-32849	1:500

Table 2. Primer sequences used for gene expression

Gene	Forward	Reverse
Tnf	5’-CCA CCA CGC TCT TCT GTC TA-3’	5’-CAC TTG GTG GTT TGC TAC GA-3
Il6	5’- CCA AGA GGT GAG TGC TTC CC-3’	5-‘CTG TTG TTC AGA CTC TCT CCCT-3’

Gene	Forward	Reverse
Cxcl2	5’-CCA ACC ACC AGG CTA CAG G -3’	5’-GCG TCA CAC TCA AGC TCT G-3’
Vegfa	5'-TTA CTG CTG TAC CTC CACC-3'	5'-ACA GGA CGG CTT GAA GATG-3'
Angpt2	5'-CAC ACT GAC CTT CCC CAA CT-3’	5'-GGA AGT CCA TGC CAT CT-3’
Ppia	5'-GTC TCC GAG CTG TTT GC-3’	5'-ATG GCG TGT AAA GTC ACC AC-3’
Il1b	5’-GAA ATG CCA CCT TTT GAC AGT G-3’	5’-TGG ATG CTC TCA TCA GGA CAG-3’
Serping1	5’-ACA GCC CCC TCT GAA TTC TT-3’	5’-GGA TGC TCT CCA AGT TGC TC-3’
Iigp1	5’-GGG GCA ATA GCT CAT TGG TA -3’	5’-ACC TCG AAG ACA TCC CCT TT-3’
H2-D1	5’-TCC GAG ATT GTA AAG CGT GAA GA-3’	5’-ACA GGG CAG TGC AGG GAT AG-3’
Ptgs2	5’-TTCAACACACTCTATCACTGGC-3’	5’-AGAAGCGTTTGCGGTACTCAT-3'
